# Supplementary material for: Itch receptor MRGPRX4 interacts with the receptor activity–modifying proteins
Source: J Biol Chem. 2023 Mar 30;299(5):104664. doi: 10.1016/j.jbc.2023.104664 (PMC10165273; doi:10.1016/j.jbc.2023.104664)
Supplement: Supporting information [file mmc1.docx]

**Supporting Information**

**Itch receptor MRGPRX4 interacts with the receptor activity-modifying proteins (RAMPs)**

Ilana B. Kotliar^#1,2^, Emilie Ceraudo^#1^, Kevin Kemelmakher-Liben^1^, Deena A. Oren^3^, Emily Lorenzen^1^, Tea Dodig-Crnković^4,†^, Mizuho Horioka-Duplix^1,‡^, Thomas Huber^1^, Jochen M. Schwenk^4^, Thomas P. Sakmar*^1,5^

^1^Laboratory of Chemical Biology and Signal Transduction, The Rockefeller University, 1230 York Ave, New York, NY 10065, USA.

^2^Tri-Institutional PhD Program in Chemical Biology, New York, NY 10065, USA

^3^Structural Biology Resource Center, The Rockefeller University, 1230 York Ave, New York, NY 10065, USA.

^4^Science for Life Laboratory, Department of Protein Science, School of Engineering Sciences in Chemistry, Biotechnology and Health, KTH Royal Institute of Technology, 171 65 Solna, Sweden.

^5^Department of Neurobiology, Care Sciences and Society, Section for Neurogeriatrics, Karolinska Institutet, 171 64 Solna, Sweden.

^#^Contributed equally.

^*^Corresponding author. Correspondence to: [Sakmar@rockefeller.edu](mailto:Sakmar@rockefeller.edu)

^†^Current location: Department of Microbiology, Public Health Agency of Sweden, Solna, Sweden.

^‡^Current location: Exscientia, The Schrödinger Building, Oxford Science Park, Oxford, UK.

**List of supporting information:**

**Supporting Figures**

Fig. S1, Chemical structures of selected MRGPRX4 agonists and IP1 dose-response curves for nateglinide and DCA as a function of MRGPRX4 expression level.

Fig. S2, Validation of mAbs to detect engineered and solubilized RAMPs and MRGPRX4 via epitope tags.­­

Fig. S3, The effect of RAMP co-expression on nateglinide and DCA dose-response curves.

Fig. S4, Characterization of the signaling properties of the different MRGPRX4, CALCRL, and RAMP constructs used in this study.

Fig. S5, Optimization of the Tet-On inducible expression system using Tet-On FLAG-NLuc-HT7-MRGPRX4-GFP10-1D4 construct.

Fig. S6, Map of interacting residues for Alpha-Fold Multimer MRGPRX4-RAMP2 complex structure.

**Supporting Tables**

Table S1. Fitting parameters of IP1 Dose-response curves in Fig. 1, Fig. 3, Fig. S4, and Fig. S5.

Table S2. Statistical test parameters for figures assessed by ordinary one-way ANOVA followed by Dunnett’s multiple comparisons test or two-tailed t test.

Table S3. Two-Phase Decay Model for Time-course best-fit values.

Table S4. MRGPRX4-RAMP2 predicted interacting residues, based on the Alpha-Fold Multimer predicted structure and the PDBePISA interface tool.

Table S5. All oligonucleotides used to generate the various constructs used in this paper.

**Source data** – provided as separate file.

**References for Supplementary Information**


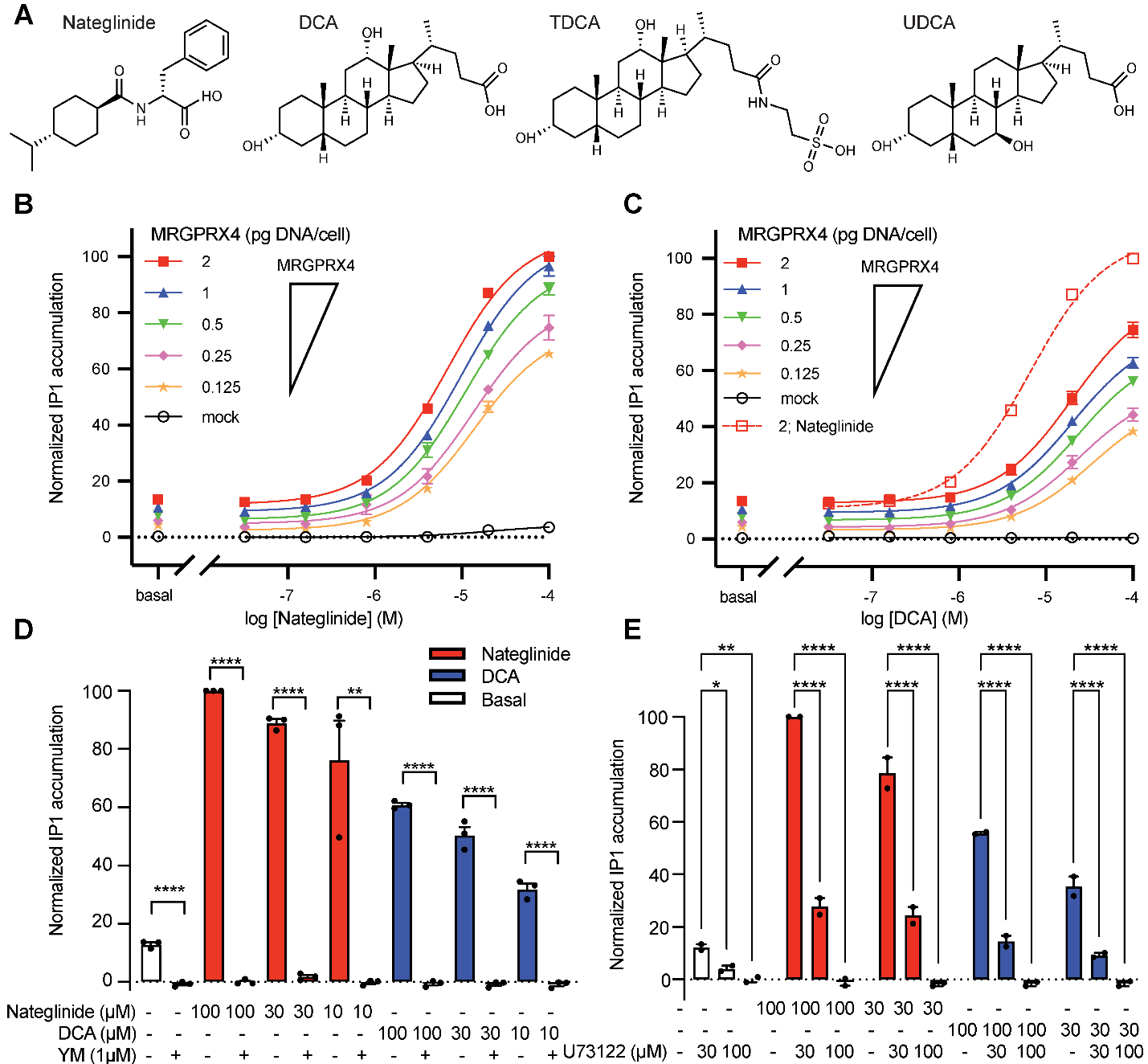


**Figure S1. Chemical structures of selected MRGPRX4 agonists and IP1 dose-response curves for nateglinide and DCA as a function of MRGPRX4 expression level.** *A,* Chemical structures for nateglinide, DCA, TDCA, and UDCA.

*B-C,* Dose-dependent IP1 accumulation for nateglinide (*B*) and DCA (*C*) in HEK293T cells transfected with five different amounts of MRGPRX4-encoding plasmid (red square, 2pg/cell; blue triangle, 1pg/cell; green reverse triangle, 0.5pg/cell; pink diamond, 0.25pg/cell; orange star, 0.125pg/cell) compared with mock-transfected cells (black open circle, 2pg/cell). The curves are fitted to log[agonist] versus response with a three-parameters model. Fitting parameters are provided in **Table S1**. Data are expressed as the mean ± SEM of the normalized IP1 accumulation to 100 µM nateglinide-induced cells transfected with 2pg/cell of MRGPRX4 plasmid as the maximum, and mock basal as the minimum. These results were used to prepare **Fig. 1B** and **C.** Data are from three independent experiments performed in four technical replicates. *D-E,* IP1 accumulation mediated by MRGPRX4 in the presence of nateglinide or DCA with or without the Gq inhibitor YM (*D*) or the phospholipase C (PLC) inhibitor U73122 (*E*). Data are expressed as the mean ± SEM of the normalized IP1 accumulation to 100 µM nateglinide and are from three (*D*) and two (*E*) independent experiments performed in four technical replicates. Datapoints representing the mean of each independent experiment are overlaid. Statistical significance was determined by unpaired two-tailed t test (for data in panel **D**) or ordinary two-way ANOVA followed by Dunnett’s multiple comparisons test (for data in panel **E**) (see **Table S2)**. Statistical significance, *****p* < 0.0001, ***p* < 0.01, * *p* < 0.1.


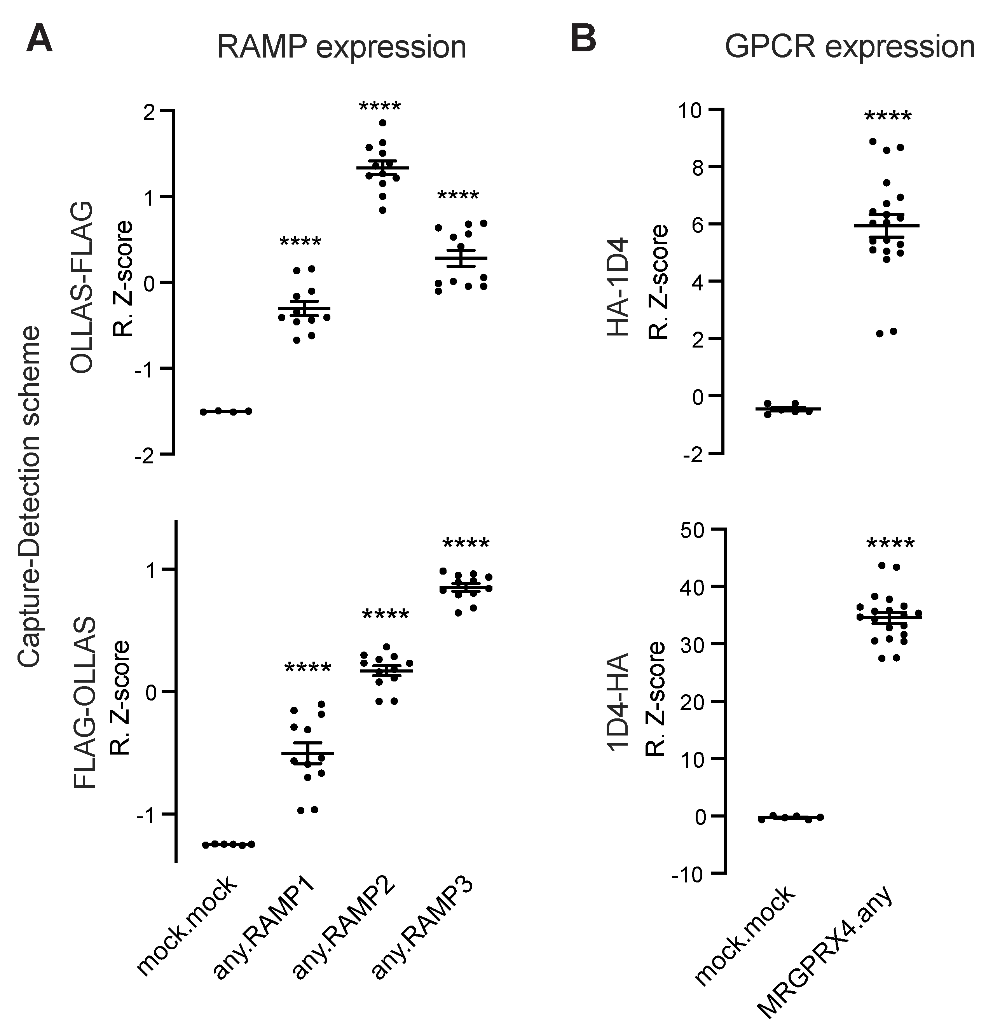


**Figure S2. Validation of mAbs to detect engineered and solubilized RAMPs and MRGPRX4 via epitope tags.** *A,* Freestyle293 cells were transfected with either epitope-tagged HA-MRGPRX4-1D4 or FLAG-RAMP-OLLAS, or co-transfected pairwise with MRGPRX4 and each RAMP. Lysates were incubated with the SBA, which included beads conjugated to each of the four mAbs targeting HA, 1D4, FLAG, and OLLAS. Each RAMP was captured with anti-OLLAS mAb beads and detected with PE-conjugated anti-FLAG mAb (*top*) and captured with anti-FLAG mAb beads and detected with PE-conjugated anti-OLLAS mAb (*bottom*). *B,* MRGPRX4 was captured with anti-HA mAb beads and detected with PE-conjugated anti-1D4 mAb (*top*) and captured with anti-1D4 mAb beads and detected with PE-conjugated anti-HA mAb (*bottom*). Data are plotted as Robust Z-scores (R.Z-scores) and represent measurements from three independent experiments performed in duplicate. Data are plotted as the mean ± SEM with individual datapoints overlaid. Asterisks indicates that signal was significantly higher than mock.mock, as determined by ordinary one-way ANOVA followed by Dunnett’s multiple comparisons test (for data in panel *A*) or unpaired two-tailed t test (for data in panel *B*) (see **Table S2** for numerical parameters) (*****p* < 0.0001).


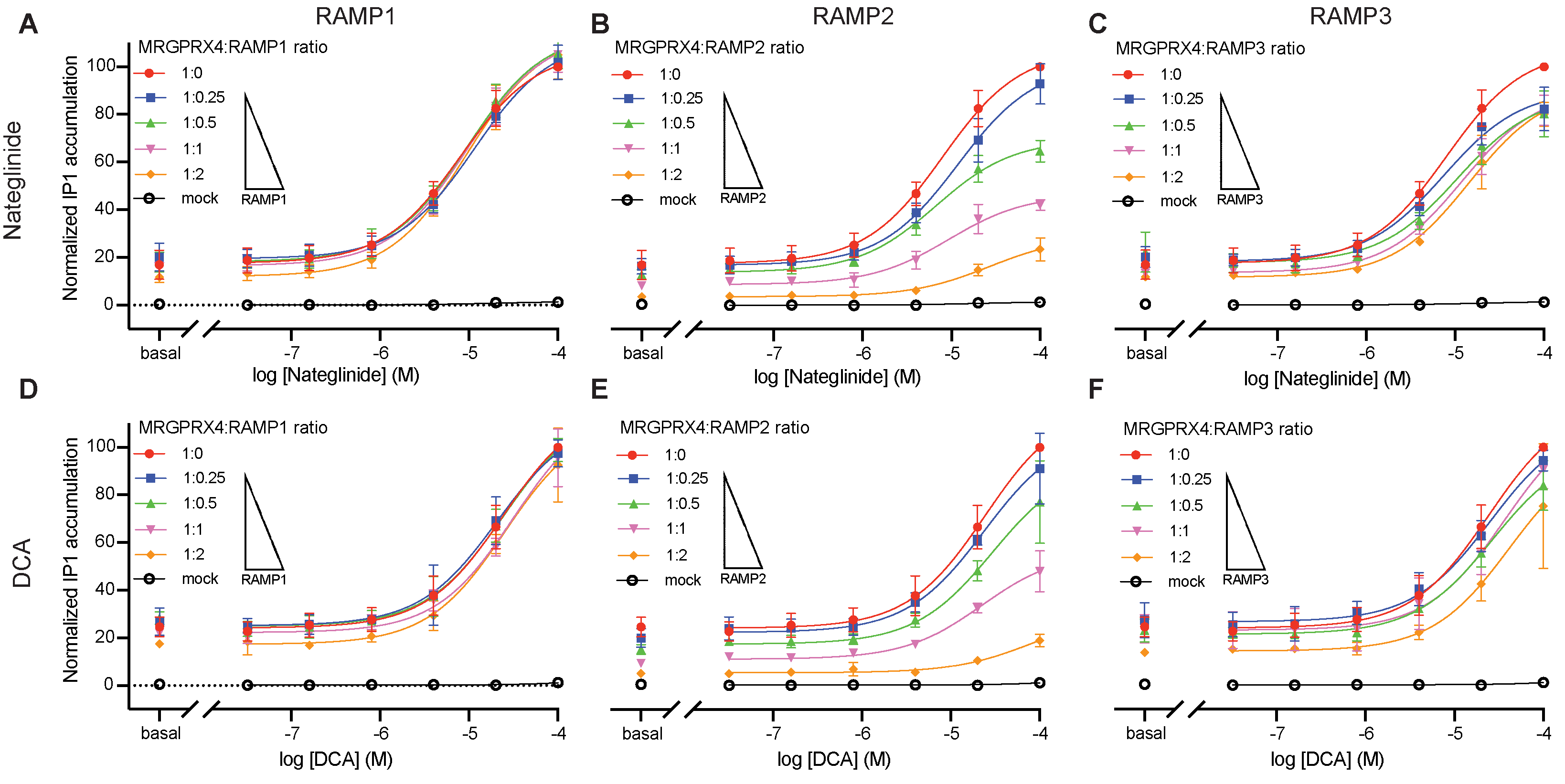


**Figure S3. The effect of RAMP co-expression on nateglinide and DCA dose-response curves.** *A-C,* IP1 accumulation was measured in response to nateglinide in cells expressing MRGPRX4 and increasing amounts of RAMP1 (*A*), RAMP2 (*B*) or RAMP3 (*C*). *D-F,* IP1 accumulation was measured in response to DCA in cells expressing MRGPRX4 and increasing amounts of RAMP1 (*D*), RAMP2 (*E*) or RAMP3 (*F*). MRGPRX4:RAMP ratios are as follows: 1:0 red circle, 1:0.25 blue square, 1:0.5 green triangle, 1:1 pink reverse triangle, 1:2 orange diamond, and mock black open circle. These results were used to prepare **Fig. 4** in the main text. The curves are fits of the dose-response data to log[agonist] versus response with a three-parameters model. Fitting parameters are provided in **Table S1**. Data are expressed as the mean ± SEM. Normalized IP1 accumulation is for each agonist separately. Data are from three independent experiments performed in four technical replicates.


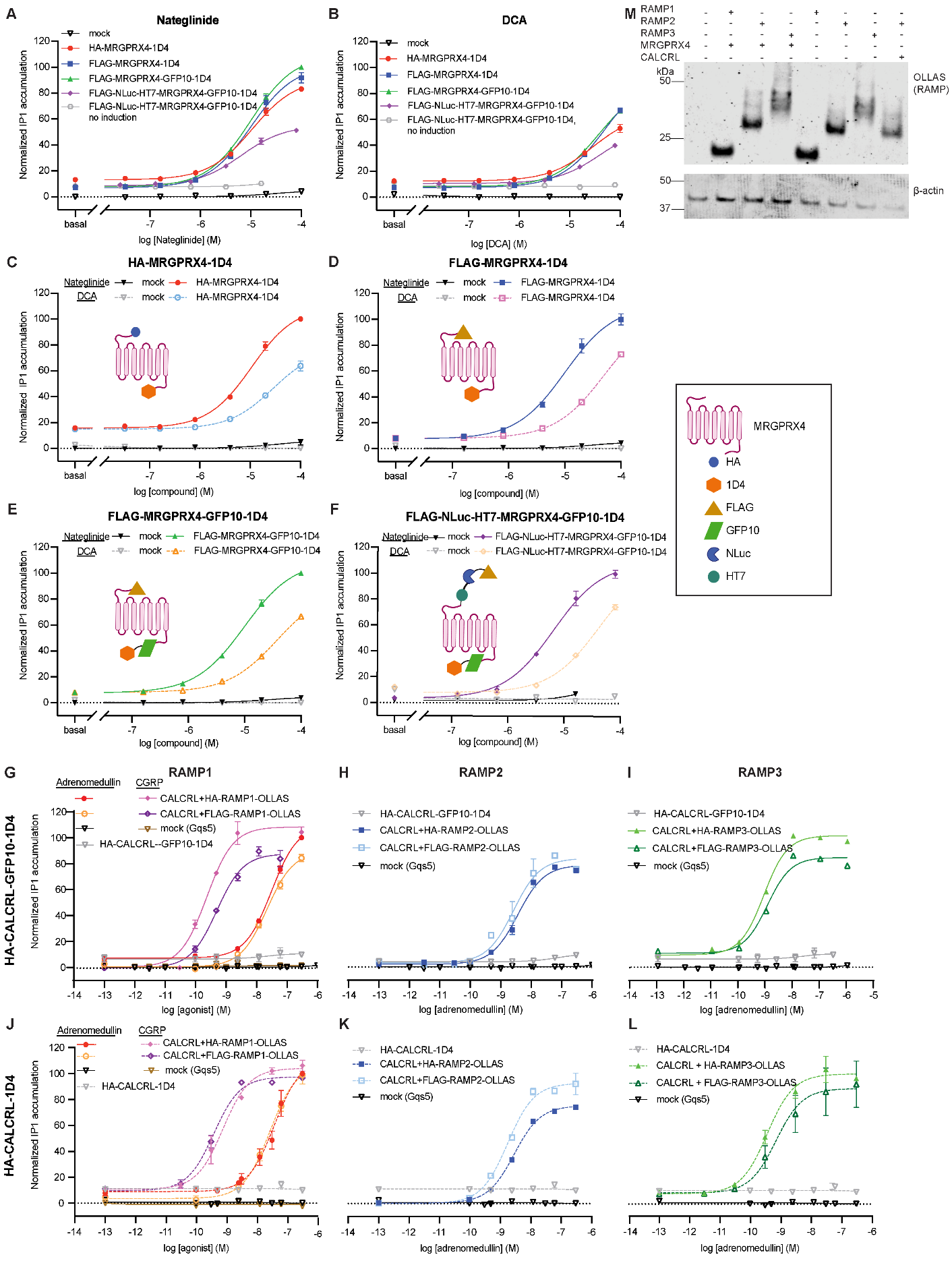


**Figure S4. Characterization of the signaling properties of the different MRGPRX4, CALCRL, and RAMP constructs used in this study**.

*A-F,* IP1 accumulation in cells expressing the different MRGPRX4 constructs used in the study. All dose-response curves are normalized to the maximum IP1 accumulation for nateglinide-stimulated FLAG-MRGPRX4-GFP10-1D4. *A-B,* IP1 accumulation dose-responses of nateglinide (*A*) and DCA (*B*) mediated by HA-MRGPRX4-1D4 (red circle), FLAG-MRGPRX4-1D4 (blue square), FLAG-MRGPRX4-GFP10-1D4 (green triangle), and Tet-On FLAG-NLuc-HT7-MRGPRX4-GFP10-1D4 with dox (purple diamond) and without dox (open gray circle). *C-F,* Dose-responses of nateglinide (filled symbol, solid line) and DCA (open symbol, dashed line) for each MRGPRX4 construct compared with mock. *C,* HA-MRGPRX4-1D4 (used in **Fig. 2, Fig. S2**), *D,* FLAG-MRGPRX4-1D4, *E,* FLAG-MRGPRX4-GFP10-1D4 (used in **Fig. 1,3,4,5, Fig. S1, Fig. S3**), and *F,* Tet-On FLAG-NLuc-HT7-MRGPRX4-GFP10-1D4 with dox compared with mock (no dox) (used in **Fig. 6, Fig. S5**). *G-L,* IP1 accumulation in cells expressing the different RAMP constructs in the presence of an epitope-tagged CALCRL. Dose-responses of adrenomedullin (all RAMPs) and calcitonin gene-related peptide (CGRP) (RAMP1 only) promoted by HA-CALCRL-GFP10-1D4 (used in **Fig. 5**) or HA-CALCRL-1D4 (used in the GPCR-RAMP interactome SBA pilot (1)) in complex with either HA-RAMP-OLLAS (used in **Fig. 3,4,5,6, Fig. S3, Fig. S5**) or FLAG-RAMP-OLLAS (used in **Fig. 2, Fig. S2** and in the GPCR-RAMP interactome SBA pilot (1)). Cells transfected with CALCLR-RAMP2 served as the positive control for RAMP functionality. IP1 accumulation is normalized to adrenomedullin stimulated FLAG-CALCRL-GFP10-1D4 + RAMP1 (*G-I*) or HA-CALCRL-1D4 + RAMP1 (*J-L*). Data are expressed as the mean ± SEM of normalized IP1 accumulation and are from three independent experiments performed in technical triplicate. Fitting parameters are in **Table S1**. *M,* OLLAS Ab was used for immunodetection of each epitope-tagged RAMP construct (HA-RAMP-OLLAS) in the presence or absence of FLAG-MRGPRX4-GFP10-1D4 or HA-CALCRL-GFP10-1D4. (Uncropped immunoblots in **Source data**).

**
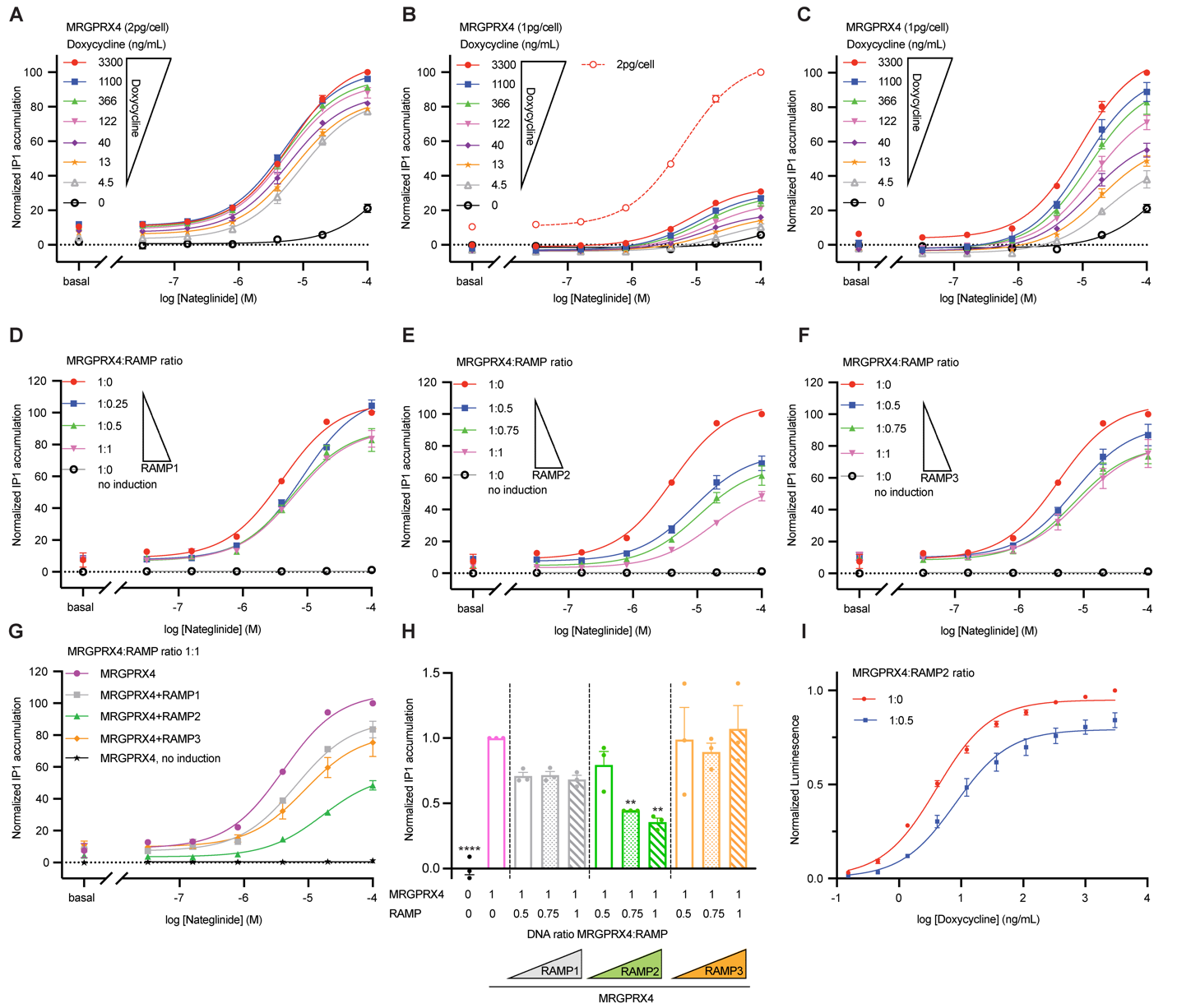
**

**Figure S5. Optimization of the Tet-On inducible expression system using Tet-On FLAG-NLuc-HT7-MRGPRX4-GFP10-1D4 construct.**

*A-C,* Three-dimensional titration of Tet-On MRGPRX4 constructs comparing receptor expression, receptor induction (dox titration with seven concentrations, up to 3300 ng/mL), and nateglinide dose-response (six concentrations and basal). Dose-response curves are normalized to IP1 accumulation in nateglinide-stimulated MRGPRX4 expressing cells ((*A,B*), 2 pg DNA/cell; (*C*), 1 pg DNA/cell). Data represent three independent experiments with four technical replicate each. *D-H,* Effect of increasing amounts of each RAMP co-expressed with Tet-On MRGPRX4 construct on nateglinide-dependent IP1 accumulation. (*D*)*,* RAMP1; (*E*)*,* RAMP2; (*F*)*,* RAMP3. *G,* Effect of Tet-On MRGPRX4-RAMP co-expression (DNA ratio 1:1) on nateglinide-dependent IP1 accumulation (data are from *D-F*). Data are normalized to nateglinide stimulated-MRGPRX4. The curves are fits of the dose-response data to log[agonist] versus response with a three-parameters model. Fitting parameters are provided in **Table S1**. *H,* Effect of increasing amounts of RAMP co-expression on basal IP1 accumulation promoted by the Tet-On MRGPRX4 construct. Datapoints representing the mean of each independent experiment are overlaid. Data are normalized to MRGPRX4 basal and represent two independent experiments with four technical replicate each. Statistical significance was determined by ordinary one-way ANOVA followed by Dunnett’s multiple comparisons test to MRGPRX4 alone (see **Table S2**) (*****p* < 0.0001, ***p* < 0.01, if not marked then not significant). *I,* Dox dose-dependent receptor total expression following induction of Tet-On MRGPRX4, expressed alone or with RAMP2 (DNA ratio 1:0.5). Data are plotted as total luminescence normalized to MRGPRX4 alone (maximal concentration of dox) and are from three independent experiments with three replicates each. Data are displayed as the mean ± SEM.


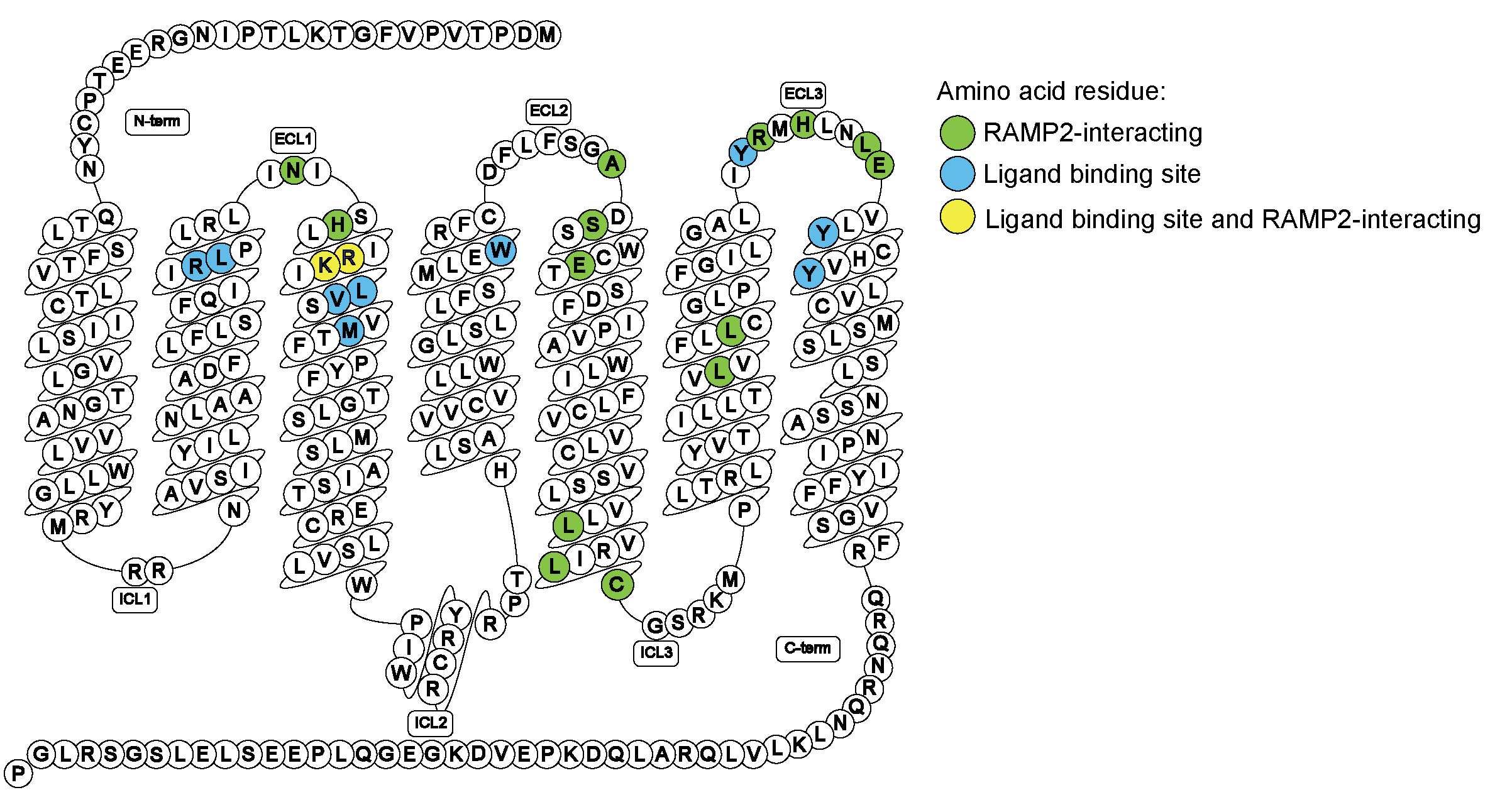


**Figure S6. Map of interacting residues for Alpha-Fold Multimer MRGPRX4-RAMP2 complex structure.** Predicted complex formation between MRGPRX4 and RAMP2 was generated with Alpha-Fold Multimer Colab (2,3). Snake plot diagram of MRGPRX4 showing the predicted RAMP2-interacting residues (green), ligand binding site residues (blue, for nateglinide analog MS47134 (4)), and ligand-binding site residues that are also predicted to interact with RAMP2 (yellow). N-term, N-terminus domain; C-term, C-terminus domain; ECL, extgracellular loop; ICL, intracellular loop. Plot generated with GPCRdb (5,6). The full list of predicted interacting residues is provided in **Table S4.**

**Table S1**. Fitting parameters of IP1 Dose-response curves in **Fig. 1, Fig. 3, Fig. S4,** and **Fig. S5**.

|  | Basal | E_max_ | logEC_50_ | EC_50_ | span | Dof ^a)^ |
| --- | --- | --- | --- | --- | --- | --- |
| MRGPRX4 (**Fig. 1A**) | | | | | | |
| Nateglinide | 14.95 | 102.6 | -4.973 | 1.065e-5 | 87.64 | 90 |
| DCA | 15.03 | 77.92 | -4.716 | 1.921e-5 | 62.89 | 85 |
| TDCA | 14.54 | 84.17 | -4.274 | 5.321e-5 | 69.63 | 77 |
| UDCA | 13.66 | 24956 | -0.9961 | 0.1009 | 24924 | 77 |
| MRGPRX4 (Dose-response Nateglinide) (**Fig.S1A**) | | | | | | |
| 2 pg | 11.77 | 108.0 | -5.168 | 6.797e-6 | 96.26 | 18 |
| 1 pg | 9.219 | 105.9 | -5.009 | 9.786e-6 | 96.66 | 18 |
| 0.5 pg | 6.421 | 97.45 | -4.958 | 1.102e-5 | 91.03 | 18 |
| 0.25 pg | 4.926 | 84.39 | -4.865 | 1.364e-5 | 79.46 | 18 |
| 0.125 pg | 2.563 | 74.80 | -4.856 | 1.392e-5 | 72.24 | 18 |
| MRGPRX4 (Dose-response DCA) (**Fig. S1B**) | | | | | | |
| 2 pg | 12.99 | 87.15 | -4.692 | 2.031e-5 | 74.16 | 18 |
| 1 pg | 9.471 | 73.82 | -4.691 | 2.039e-5 | 64.35 | 18 |
| 0.5 pg | 6.783 | 68.02 | -4.622 | 2.389e-5 | 61.23 | 18 |
| 0.25 pg | 4.249 | 54.24 | -4.609 | 2.461e-5 | 49.99 | 18 |
| 0.125 pg | 3.308 | 50.32 | -4.470 | 3.390e-5 | 47.01 | 18 |
| MRGPRX4 + titration RAMP1 (Dose-response Nateglinide) (**Fig. S3A**) | | | | | | |
| 2 pg (of RAMP1) | 11.98 | 114.4 | -5.028 | 9.375e-6 | 102.4 | 18 |
| 1 pg | 16.52 | 113.9 | -5.025 | 9.448e-6 | 97.37 | 18 |
| 0.5 pg | 18.21 | 114.8 | -5.019 | 9.564e-6 | 96.60 | 18 |
| 0.25 pg | 19.44 | 111.9 | -4.939 | 1.152e-5 | 92.43 | 18 |
| MRGPRX4 + titration RAMP2 (Dose-response Nateglinide) (**Fig. S3B**) | | | | | | |
| 2 pg | 3.623 | 28.83 | -4.574 | 2.664e-5 | 25.21 | 18 |
| 1 pg | 8.724 | 46.54 | -5.032 | 9.299e-5 | 37.81 | 17 |
| 0.5 pg | 13.85 | 69.53 | -5.175 | 6.685e-5 | 55.69 | 17 |
| 0.25 pg | 16.83 | 101.3 | -4.928 | 1.181e-5 | 84.47 | 17 |
| MRGPRX4 + titration RAMP3 (Dose-response Nateglinide) (**Fig. S3C**) | | | | | | |
| 2 pg | 11.70 | 91.35 | -4.839 | 1.450e-5 | 79.65 | 17 |
| 1 pg | 13.65 | 90.43 | -4.920 | 1.202e-5 | 76.78 | 17 |
| 0.5 pg | 17.78 | 88.29 | -4.977 | 1.055e-5 | 70.51 | 18 |
| 0.25 pg | 18.35 | 90.32 | -5.128 | 7.440e-6 | 71.98 | 17 |
| MRGPRX4 + titration RAMP1 (Dose-response DCA) (**Fig. S3D**) | | | | | | |
| 2 pg | 17.30 | 111.8 | -4.597 | 2.532e-5 | 94.50 | 18 |
| 1 pg | 22.25 | 118.0 | -4.504 | 3.136e-5 | 95.79 | 18 |
| 0.5 pg | 24.94 | 117.0 | -4.615 | 2.424e-5 | 92.02 | 18 |
| 0.25 pg | 25.16 | 112.8 | -4.683 | 2.077e-5 | 87.65 | 18 |
| MRGPRX4 + titration RAMP2 (Dose-response DCA) (**Fig. S3E**) | | | | | | |
| 2 pg | 5.412 | 29.86 | -4.097 | 8.007e-5 | 24.45 | 20 |
| 1 pg | 11.18 | 56.18 | -4.652 | 2.226e-5 | 44.99 | 20 |
| 0.5 pg | 17.50 | 94.46 | -4.530 | 2.952e-5 | 76.96 | 20 |
| 0.25 pg | 22.40 | 107.2 | -4.625 | 2.371e-5 | 84.83 | 20 |
| MRGPRX4 + titration RAMP3 (Dose-response DCA) (**Fig. S3F**) | | | | | | |
| 2 pg | 14.52 | 100.4 | -4.384 | 4.128e-5 | 85.85 | 18 |
| 1 pg | 23.28 | 115.4 | -4.436 | 3.667e-5 | 92.07 | 18 |
| 0.5 pg | 21.60 | 100.4 | -4.574 | 2.664e-5 | 78.85 | 18 |
| 0.25 pg | 26.80 | 111.0 | -4.596 | 2.533e-5 | 84.19 | 18 |
| MRGPRX4 (compare constructs) (Dose-response Nateglinide) (**Fig. S4A,C-F**) | | | | | | |
| HA-MRGPRX4-1D4 | 13.13 | 91.49 | -4.972 | 1.066e-5 | 78.36 | 18 |
| FLAG-MRGPRX4-1D4 | 6.903 | 102.0 | -4.994 | 1.015e-5 | 95.07 | 18 |
| FLAG-MRGPRX4-GFP10-1D4 | 7.722 | 109.7 | -5.004 | 9.908e-6 | 101.9 | 18 |
| FLAG-NLuc-HT7-MRGPRX4-GFP10-1D4 | 8.575 | 55.22 | -5.193 | 6.407e-6 | 46.65 | 18 |
| MRGPRX4 (compare constructs) (Dose-response DCA) (**Fig. S4B,C-F**) | | | | | | |
| Mock | 2.330 | 0.2364 | -7.834 | 1.465e-8 | -2.094 | 18 |
| HA-MRGPRX4-1D4 | 12.52 | 64.71 | -4.544 | 2.855e-5 | 52.19 | 18 |
| FLAG-MRGPRX4-1D4 | 7.469 | 96.26 | -4.308 | 4.920e-5 | 88.79 | 18 |
| FLAG-MRGPRX4-GFP10-1D4 | 8.165 | 88.54 | -4.424 | 3.764e-5 | 80.37 | 18 |
| FLAG-NLuc-HT7-MRGPRX4-GFP10-1D4 | 10.56 | 55.05 | -4.388 | 4.093e-5 | 44.49 | 18 |
| HA-CALCRL-GFP10-1D4 (Dose-response Adrenomedullin) (**Fig. S4G-I**) | | | | | | |
| Mock | 0.1993 | 11.54 | -5.176 | 6.665e-6 | 11.34 | 42 |
| CALCRL | 4.246 | 10.49 | -7.129 | 7.435e-8 | 6.243 | 11 |
| CALCRL+HA-RAMP1-OLLAS | 7.282 | 109.6 | -7.512 | 3.074e-008 | 102.4 | 17 |
| CALCRL+FLAG-RAMP1-OLLAS | 0.4611 | 89.41 | -7.655 | 2.213e-008 | 88.95 | 18 |
| CALCRL+HA-RAMP2-OLLAS | 2.278 | 78.81 | -8.436 | 3.665e-9 | 76.54 | 11 |
| CALCRL+FLAG-RAMP2-OLLAS | 3.344 | 83.87 | -8.581 | 2.626e-9 | 80.53 | 10 |
| CALCRL+HA-RAMP3-OLLAS | 9.590 | 101.6 | -9.089 | 8.142e-010 | 92.04 | 17 |
| CALCRL+FLAG-RAMP3-OLLAS | 11.09 | 84.86 | -8.955 | 1.109e-009 | 73.76 | 18 |
| HA-CALCRL-GFP10-1D4 (Dose-response CGRP) (**Fig. S4G**) | | | | | | |
| Mock | 0.4062 | 1.291 | -9.835 | 1.461e-10 | 0.8853 | 41 |
| CALCRL | 6.580 | 10.82 | -7.655 | 2.212e-8 | 4.236 | 18 |
| CALCRL+HA-RAMP1-OLLAS | 0.3849 | 105.5 | -9.635 | 2.317e-010 | 105.1 | 12 |
| CALCRL+FLAG-RAMP1-OLLAS | -0.3006 | 87.64 | -9.305 | 4.957e-010 | 87.94 | 9 |
| HA-CALCRL-1D4 (Dose-response Adrenomedullin) (**Fig. S4J-L**) | | | | | | |
| Mock | 0.9133 | 0.05397 | -7.376 | 4.210e-008 | -0.8593 | 30 |
| CALCRL | 11.35 | Unstable | -0.7361 | 0.1836 | Unstable | 30 |
| CALCRL+HA-RAMP1-OLLAS | 9.024 | 111.5 | -7.444 | 3.599e-008 | 102.5 | 30 |
| CALCRL+FLAG-RAMP1-OLLAS | 3.676 | 106.5 | -7.596 | 2.538e-008 | 102.9 | 27 |
| CALCRL+HA-RAMP2-OLLAS | 0.3834 | 75.53 | -8.549 | 2.827e-009 | 75.15 | 18 |
| CALCRL+FLAG-RAMP2-OLLAS | 0.1775 | 92.88 | -8.758 | 1.746e-009 | 92.70 | 17 |
| CALCRL+HA-RAMP3-OLLAS | 8.590 | 108.1 | -9.450 | 3.544e-010 | 99.49 | 9 |
| CALCRL+FLAG-RAMP3-OLLAS | 9.152 | 95.99 | -9.162 | 6.881e-010 | 86.83 | 11 |
| HA-CALCRL- 1D4 (Dose-response CGRP) (**Fig. S4J**) | | | | | | |
| Mock | Unstable | -0.8959 | -16.10 | 7.881e-017 | Unstable | 24 |
| CALCRL+HA-RAMP1-OLLAS | 10.86 | 104.1 | -9.130 | 7.412e-010 | 93.21 | 9 |
| CALCRL+FLAG-RAMP1-OLLAS | 9.681 | 97.50 | -9.435 | 3.673e-010 | 87.81 | 9 |
| FLAG-NLuc-HT7-MRGPRX4-GFP10-1D4 (Titration doxycycline) (2pg/cell) (**Fig. S5A**) | | | | | | |
| Dox 3300 ng/mL | 10.81 | 106.4 | -5.197 | 6.360e-6 | 95.60 | 18 |
| Dox 1100 ng/mL | 11.03 | 101.4 | -5.267 | 5.302e-6 | 90.38 | 18 |
| Dox 366 ng/mL | 9.980 | 97.01 | -5.274 | 5.320e-6 | 87.03 | 18 |
| Dox 122 ng/mL | 9.245 | 94.00 | -5.268 | 5.400e-6 | 84.76 | 18 |
| Dox 40 ng/mL | 7.421 | 87.55 | -5.217 | 6.065e-6 | 80.13 | 18 |
| Dox 13 ng/mL | 6.149 | 84.94 | -5.134 | 7.354e-6 | 78.79 | 18 |
| Dox 4.5 ng/mL | 3.549 | 84.60 | -5.040 | 9.121e-6 | 81.05 | 18 |
| No dox | 0.7169 | 72.26 | -3.602 | 0.0002500 | 71.55 | 18 |
| FLAG-NLuc-HT7-MRGPRX4-GFP10-1D4 (Titration doxycycline) (1pg/cell) (**Fig. S5B**) | | | | | | |
| Dox 3300 ng/mL | -1.008 | 34.37 | -5.033 | 9.258e-6 | 35.37 | 18 |
| Dox 1100 ng/mL | -3.039 | 30.65 | -4.968 | 1.077e-5 | 33.69 | 18 |
| Dox 366 ng/mL | -3.000 | 29.08 | -4.893 | 1.279e-5 | 32.08 | 18 |
| Dox 122 ng/mL | -2.879 | 24.93 | -4.821 | 1.510e-5 | 27.81 | 18 |
| Dox 40 ng/mL | -3.380 | 18.76 | -4.862 | 1.373e-5 | 22.14 | 18 |
| Dox 13 ng/mL | -3.433 | 17.37 | -4.718 | 1.915e-5 | 20.80 | 18 |
| Dox 4.5 ng/mL | -3.809 | 14.10 | -4.590 | 2.568e-5 | 17.91 | 18 |
| No dox | -1.503 | 24.99 | -3.577 | 0.0002650 | 26.49 | 18 |
| FLAG-NLuc-HT7-MRGPRX4-GFP10-1D4 (Titration doxycycline) (over 1pg/cell Normalized) (**Fig. S5C**) | | | | | | |
| Dox 3300 ng/mL | 3.818 | 110.7 | -5.036 | 9.197e-6 | 106.8 | 18 |
| Dox 1100 ng/mL | -2.284 | 99.95 | -4.969 | 1.073e-5 | 102.2 | 18 |
| Dox 366 ng/mL | -2.161 | 94.31 | -4.900 | 1.258e-5 | 96.48 | 18 |
| Dox 122 ng/mL | -1.806 | 82.37 | -4.824 | 1.499e-5 | 84.18 | 18 |
| Dox 40 ng/mL | -3.289 | 63.50 | -4.868 | 1.355e-5 | 66.79 | 18 |
| Dox 13 ng/mL | -3.443 | 59.23 | -4.721 | 1.901e-5 | 62.67 | 18 |
| Dox 4.5 ng/mL | -4.623 | 48.88 | -4.601 | 2.506e-5 | 53.50 | 18 |
| No dox | -1.726 | 55.58 | -3.825 | 0.0001497 | 57.31 | 18 |
| FLAG-NLuc-HT7-MRGPRX4-GFP10-1D4 + RAMP1 (Titration RAMP1) (**Fig. S5D**) | | | | | | |
| Ratio 1:0 | 8.926 | 106.7 | -5.392 | 4.059e-006 | 97.74 | 11 |
| Ratio 1:0.5 | 7.772 | 111.3 | -5.074 | 8.425e-006 | 103.5 | 25 |
| Ratio 1:0.75 | 6.875 | 90.02 | -5.225 | 5.950e-006 | 83.14 | 25 |
| Ratio 1:1 | 7.220 | 89.56 | -5.192 | 6.430e-006 | 82.34 | 25 |
| Ratio 1:0, no dox | 0.2822 | 2.457 | -3.895 | 0.0001274 | 2.175 | 21 |
| FLAG-NLuc-HT7-MRGPRX4-GFP10-1D4 + RAMP2 (Titration RAMP2) (**Fig. S5E**) | | | | | | |
| Ratio 1:0 | 8.926 | 106.7 | -5.392 | 4.059e-006 | 97.74 | 11 |
| Ratio 1:0.5 | 7.610 | 75.64 | -5.058 | 8.757e-006 | 68.03 | 25 |
| Ratio 1:0.75 | 4.862 | 67.94 | -4.977 | 1.053e-005 | 63.08 | 11 |
| Ratio 1:1 | 3.554 | 55.74 | -4.778 | 1.668e-005 | 52.19 | 25 |
| Ratio 1:0, no dox | 0.2822 | 2.457 | -3.895 | 0.0001274 | 2.175 | 21 |
| FLAG-NLuc-HT7-MRGPRX4-GFP10-1D4 + RAMP3 (Titration RAMP3) (**Fig. S5F**) | | | | | | |
| Ratio 1:0 | 8.926 | 106.7 | -5.392 | 4.059e-006 | 97.74 | 11 |
| Ratio 1:0.5 | 10.08 | 93.43 | -5.144 | 7.170e-006 | 83.35 | 25 |
| Ratio 1:0.75 | 8.392 | 80.54 | -5.148 | 7.112e-006 | 72.15 | 24 |
| Ratio 1:1 | 9.885 | 81.18 | -5.056 | 8.795e-006 | 71.30 | 25 |
| Ratio 1:0, no dox | 0.2822 | 2.457 | -3.895 | 0.0001274 | 2.175 | 21 |
| FLAG-NLuc-HT7-MRGPRX4-GFP10-1D4 Basal IP1(Titration RAMPs) (**Fig. S5H**) | | | | | | |
| Ratio 1:0 | 1±0.000 | NA | NA | NA | NA | NA |
| RAMP1 Ratio 1:0.5 | 0.709±0.029 | NA | NA | NA | NA | NA |
| RAMP1 Ratio 1:75 | 0.716±0.029 | NA | NA | NA | NA | NA |
| RAMP1 Ratio 1:1 | 0.683±0.032 | NA | NA | NA | NA | NA |
| RAMP2 Ratio 1:0.5 | 0.794±0.104 | NA | NA | NA | NA | NA |
| RAMP2 Ratio 1:75 | 0.444±0.0007 | NA | NA | NA | NA | NA |
| RAMP2 Ratio 1:1 | 0.356±0.031 | NA | NA | NA | NA | NA |
| RAMP3 Ratio 1:0.5 | 0.990±0.246 | NA | NA | NA | NA | NA |
| RAMP3 Ratio 1:75 | 0.893±0.069 | NA | NA | NA | NA | NA |
| RAMP3 Ratio 1:1 | 1.072±0.179 | NA | NA | NA | NA | NA |
| FLAG-NLuc-HT7-MRGPRX4-GFP10-1D4 total expression (Titration dox) (**Fig. S5I**) | | | | | | |
| MRGPRX4 | 0.009273 | 0.9497 | 0.6070 | 4.046 | 0.9404 | 87 |
| MRGPRX4+RAMP2 | 0.003719 | 0.7930 | 0.9067 | 8.067 | 0.7893 | 87 |

a) Degrees of Freedom,

**Table S2.** Statistical test parameters for figures assessed by ordinary one-way ANOVA followed by Dunnett’s multiple comparisons test or two-tailed t test.

| Figure | Panel | DoF | F | Condition used for comparison | Notes |
| --- | --- | --- | --- | --- | --- |
| 1 | B (top) | 20 | 157.8 | MRGPRX4 (2pg/cell), for each agonist separately |  |
|  | B (bottom) | 24 | 243.3 |  |  |
| S1 | D | 4 | 2.623 | Basal | +/- YM for each condition. Unpaired, two-tailed t test. t-value = 15.57 |
|  | D | 4 | Infinity | Ng 100µM | Unpaired, two-tailed t test. t-value = 205.2 |
|  | D | 4 | 4.143 | Ng 30µM | Unpaired, two-tailed t test. t-value = 59.00 |
|  | D | 4 | 641.3 | Ng 10µM | Unpaired, two-tailed t test. t-value = 5.711 |
|  | D | 4 | 1.496 | DCA 100µM | Unpaired, two-tailed t test. t-value = 65.85 |
|  | D | 4 | 22.08 | DCA 30µM | Unpaired, two-tailed t test. t-value = 17.86 |
|  | D | 4 | 8.605 | DCA 10µM | Unpaired, two-tailed t test. t-value = 16.87 |
|  | E | 15 | 60.56, 823.1, 121.7 | No U73122 treatment | F values correspond to interaction, row factor, column factor |
| 2 | B (Left) | 22 | 269.1 | mock.mock (all) | Figure 2 Panel B LHS, top to bottom, 1D4 capture, FLAG detection |
|  | B (Left) | 28 | 197.8 |  | 1D4 capture, OLLAS detection |
|  | B (Left) | 35 | 55.03 |  | HA capture, FLAG detection |
|  | B (Left) | 35 | 46.56 |  | HA capture, OLLAS detection |
|  | B (Right) | 36 | 143.4 |  | Figure 2 Panel B RHS, top to bottom, FLAG capture, 1D4 detection |
|  | B (Right) | 31 | 258.3 |  | FLAG capture, HA detection |
|  | B (Right) | 34 | 113.4 |  | OLLAS capture, 1D4 detection |
|  | B (Right) | 30 | 71.16 |  | OLLAS capture, HA detection |
| S2 | A (top) | 35 | 123.5 | mock.mock (all) | OLLAS capture, FLAG detection |
|  | A (bottom) | 38 | 204.8 |  | FLAG capture, OLLAS detection |
|  | B (top) | 24 | 124.8 |  | HA capture, 1D4 detection. Unpaired, two-tailed t test. t-value = 8.689. |
|  | B (bottom) | 24 | 203.9 |  | 1D4 capture, HA detection. Unpaired, two-tailed t test. t-value = 19.42. |
| 3 | B | 55 | 25.79 | Mock |  |
| 4 | A (top) | 51 | 83.69 | MRGPRX4 1:0 | nateglinide or DCA- stimulated |
|  | A (bottom) | 54 | 26.24 | MRGPRX4 1:0 | MRGPRX4 1:0 |
|  | B  C  D  E  F | 64  14  14  14  14 | 40.94  82.92  71.03  83.63  57.25 | MRGPRX4 1:0  MRGPRX4 1:0  MRGPRX4 1:0  MRGPRX4 1:0  MRGPRX4 1:0 | MRGPRX4 1:0 basal  MRGPRX4,1:0+nateglinide100µM  MRGPRX4,1:0+DCA100µM  MRGPRX4,1:0+TDCA100µM  MRGPRX4,1:0+UDCA100µM |
| 5 | D | 52 | 1.854 | Basal (all) | β-arrestin1 + MRGPRX4: basal versus 100µM nateglinide Unpaired, two-tailed t test. t-value = 2.313 |
|  | D | 52 | 7.531 |  | β-arrestin2 + MRGPRX4: basal versus 100µM nateglinide Unpaired, two-tailed t test. t-value = 26.04 |
|  | D | 52 | 7.448 |  | β-arrestin1 + CALCRL + RAMP2: basal versus 200nM adrenomedullin Unpaired, two-tailed t test. t-value = 14.25 |
|  | D | 52 | 8.583 |  | β-arrestin2 + CALCRL + RAMP2: basal versus 200nM adrenomedullin Unpaired, two-tailed t test. t-value = 15.59 |
|  | F | 15 | 2.801 |  | Basal |
|  | F | 45 | 33.51 |  | Nateglinide |
|  | F | 15 | 4.4 |  | DCA |
| 6 | B | 32 | 44.67 | MRGPRX4 alone (+dox) |  |
|  | C | 26 | 4.355 | MRGPRX4 alone | Normalized BRET Ratio (left axis) |
|  | C | 26 | 5.792 |  | Surface expression (right axis) |
| S5 | H | 22 | 9.979 | MRGPRX4 alone |  |

**Table S3.** Two-Phase Decay Model for Time-course best-fit values.

|  | *y_0_* | *x_0_* | Plateau | K_fast_ | K_slow_ | Dof ^a)^ | Half-life (slow) | Half-life (fast) | Tau (slow) | Tau (fast) | Rate constant ratio |
| --- | --- | --- | --- | --- | --- | --- | --- | --- | --- | --- | --- |
| MRGPRX4-GFP10 BRET^2^ time-course (**Fig. 5**) | | | | | | |  |  |  |  |  |
| 100 µM^b)^ | 0.01291 | 56.70 | 0.004639 | 0.008519 | 0.002534 | 64 | 273.5 | 81.37 | 394.6 | 117.4 | 3.361 |
|  |  |  |  |  |  |  |  |  |  |  |  |
| CALCRL-GFP10 + RAMP2 BRET^2^ time-course (**Fig. 5**) | | | | | | | |  |  |  |  |
| 200 nM^c)^ | 0.02190 | -468.5 | -0.03173 | 0.005142 | 0.005142 | 41 | 134.8 | 134.8 | 194.5 | 194.5 | 1.000 |
|  |  |  |  |  |  |  |  |  |  |  |  |

1. Degrees of Freedom
2. Nateglinide
3. Adrenomedullin

**Table S4.** MRGPRX4-RAMP2 predicted interacting residues, based on the Alpha-Fold Multimer predicted structure and PDBePISA interface tool (7) [https://www.ebi.ac.uk/msd-srv/prot_int/cgi-bin/piserver]. The interaction types are based on manual annotation with ChimeraX (8,9). Model confidence indicates the predicted local distance difference test (pLDDT) value for each residue. pLDDT values ≥ 50 are underlined.

| MRGPRX4 residue | MRGPRX4 residue location ^a)^ | RAMP2 residue | Interaction type | MRGPRX4 model confidence | RAMP2 model confidence |
| --- | --- | --- | --- | --- | --- |
| Extracellular Interactions | | | | | |
| ASN89 | ECL1 | ASP66 | H bond | 42.4 | 67.7 |
| ASN89 | ECL1 | LEU67 | H bond (main chain) | 42.4 | 71.1 |
| ASN89 | ECL1 | GLU63 | H bond (main chain) | 42.4 | 70.8 |
| HIS92 | 3.22^TM3^ | GLU63 | H bond | 50.1 | 70.8 |
| HIS92 | 3.22^TM3^ | ASP56 | H bond | 50.1 | 72.5 |
| HIS92 | 3.22^TM3^ | GLU59 | H bond | 50.1 | 73.7 |
| ARG95 | 3.25^TM3^ | GLU59 | Salt bridge | 63.6 | 73.7 |
| ARG95 | 3.25^TM3^ | GLU63 | Salt bridge | 63.6 | 70.8 |
| LYS96 | 3.26^TM3^ | ASP56 | Salt bridge | 64.7 | 72.5 |
| ALA168 | ECL2 | ARG49 | H bond | 47.9 | 68.6 |
| SER170 | 5.29^TM5^ | ARG49 | van der Waals | 52.3 | 68.6 |
| GLU174 | 5.33^TM5^ | TRP44 | H bond | 64.3 | 66.6 |
| GLU174 | 5.33^TM5^ | ALA45 | H bond (main chain) | 64.3 | 61.4 |
| ARG241 | ECL3 | TRP44 | H bond (main chain) | 49.1 | 66.6 |
| HIS243 | ECL3 | TRP44 | Clash | 37.1 | 66.6 |
| HIS243 | ECL3 | TYR51 | Edge to face | 37.1 | 74.6 |
| HIS243 | ECL3 | HIS82 | H bond | 37.1 | 71.8 |
| LEU246 | ECL3 | TYR51 | van der Waals | 40.9 | 74.6 |
| LEU246 | ECL3 | ARG55 | van der Waals | 40.9 | 76.4 |
| GLU247 | ECL3 | PRO70 | van der Waals | 41.6 | 62.9 |
| Transmembrane Interactions | | | | | |
| Leu199 | 5.58^TM5^ | Ile118 | van der Waals | 83.4 | 48.6 |
| Leu199 | 5.58^TM5^ | Val122 | van der Waals | 83.4 | 48.3 |
| Leu203 | 5.62^TM5^ | Leu121 | van der Waals | 83.4 | 52.6 |
| Leu203 | 5.62^TM5^ | Val122 | van der Waals | 76.5 | 48.3 |
| Leu203 | 5.62^TM5^ | Arg125 | H bond | 76.5 | 51.8 |
| Cys204 | 5.63^TM5^ | Arg125 | H bond | 74.2 | 51.8 |
| Leu223 | 6.42^TM6^ | Ile115 | van der Waals | 91.9 | 44.5 |
| Leu223 | 6.42^TM6^ | Ile119 | van der Waals | 91.9 | 47.4 |
| Leu223 | 6.42^TM6^ | Ile118 | van der Waals | 91.9 | 48.6 |
| Leu227 | 6.46^TM6^ | Ile112 | van der Waals | 90.6 | 45.4 |
| Leu227 | 6.46^TM6^ | Ile115 | van der Waals | 90.6 | 44.5 |

1. MRGPRX4 residues are numbered according to the Ballesteros–Weinstein numbering obtained from GPCRdb.

**Table S5. All oligonucleotides used to generate the various constructs used in this paper.** *5A,* TagMaster site-directed mutagenesis primers used to swap the FLAG epitope tag with a 3xHA epitope tag in FLAG-RAMP-OLLAS constructs. *5B,* NEBuilder HiFi DNA Assembly primers used to amplify fragments and build the GPCR constructs used in this study. All oligonucleotides were ordered from IDT at the standard desalting grade.

*5A,* Primers for replacement of FLAG epitope tag with 3x HA tag for RAMPs

| Purpose of Primer | Oligonucleotide Sequence of Primers |
| --- | --- |
| RAMP1 Forward | CTG TTT ATG ACC ACC GCC TAC CCA TAC |
| RAMP1 Reverse | TAG TTG GCT TCC TGC AAT TGG CAA GCG |
| RAMP2 Forward | CCT CAC GAG GCC CTG GCC TAC CCA TA |
| RAMP2 Reverse | AGG CAG GGG CTG CAA TTG AGC GTA AT |
| RAMP3 Forward | TGT CCT AGA GCC GGC GGA TAC CCA TA |
| RAMP3 Reverse | CCT GTC TCG TTG CAC AAT TGA GCG TAA |

*5B,* NEBuilder HiFi DNA Assembly primers used in building constructs as described in Methods. Primers are grouped by construct generated (lines within table).

| Direction | Sequence of Primers (5’ to 3’) w/ overlaps underlined | Purpose |
| --- | --- | --- |
| Forward (SP-FLAG-MRGPRX4) | CGT TTA AAC TTA AGC TTA GCG CCA CCA TGA AGA CGA TCA TC | Used in building FLAG-MRGPRX4-1D4 from PRESTO-Tango MRGPRX4 for PLA assay (1D4-pcDNA 3.1(+) vector was subject to enzymatic double digestion and purification). |
| Reverse (SP-FLAG-MRGPRX4) | TGC TGG CCT CAT CGA ATT CAC CGG TGC GTC CAC CGG TAT C |  |
| Forward (SP-FLAG-MRGPRX4) | CTG CAC GAG ATG ATA CCG CCG CCA CCA TGA AGA CGA TC | Used in building FLAG-MRGPRX4-GFP10-1D4 for BRET^2^ and IP_1_ assays |
| Reverse (SP-FLAG-MRGPRX4) | CTC ACG AAT TCA CCG GTA CCG AAT TCA CCG GTG CGT CC |  |
| Forward (GFP10-1D4 pcDNA 3.1(+) vector) | GGT ACC GGT GAA TTC GTG |  |
| Reverse (GFP10-1D4 pcDNA 3.1(+) vector) | GGC GGT ATC ATC TCG TGC |  |
| Forward (SP-FLAG-CALCRL) | CTG CAC GAG ATG ATA CCG CCG CCA CCA TGC GGC TGT GC | Used in building SP-FLAG-CALCRL-GFP10-1D4 |
| Reverse (SP-FLAG-CALCRL) | CTC ACG AAT TCA CCG GTA CCG GTA CCG TTG TAC AGA TTC TCG GG | Note: Forward and Reverse (GFP10-1D4 pcDNA 3.1(+) vector) primers as the same as above |
| Forward (MRGPRX4-GFP10) | CCC CTG CAG GAG ATG ACA CCA TGG ATC CTA CTG TGC CTG TG | Used in building Tet-On SP-FLAG-Nluc-HT7-MRGPRX4-GFP10-1D4 for surface expression assays |
| Reverse (MRGPRX4-GFP10) | ACG GTG GTG CTG GCC TCA TCG GAT CCG CCT GCA GGC TT |  |
| Forward (SP-FLAG-Nluc-HT7) | TTT GTA CAA AAA AGC AGG CTC CCA AGC TGG CTA GCG TTT AAA C |  |
|  |  |  |
| Reverse (SP-FLAG-Nluc-HT7) | GGT GTC ATC TCC TGC AGG |  |
| Forward (1D4 Tet-On vector) | GAT GAG GCC AGC ACC ACC |  |
| Reverse (1D4 Tet-On vector) | AGC CTG CTT TTT TGT ACA AAC TTG C |  |
| Forward (CALCRL-GFP10) | CCC CTG CAG GAG ATG ACA CCG AGC TGG AAG AGA GCC CC | Used in building Tet-On SP-FLAG-Nluc-HT7-CALCRL-GFP10-1D4 for surface expression assays |
| Reverse (CALCRL-GFP10) | ACG GTG GTG CTG GCC TCA TCC TTG TAC AGC TCG TCC ATG C | Note: Forward and Reverse (SP-FLAG-Nluc-HT7) and Forward and Reverse (1D4 Tet-On vector) primers are the same as above |

**Supplementary References**

Note: In the main text references, Lorenzen et al., is reference 13, Jumper et al., is reference 16, Evans et al., is reference 17, Cao et al., is reference 16, Isberg et al., is reference 67, Pandy-Szekeres et al., is reference 68, Krissinel et al., is reference 66, Goddard et al., is reference 21, and Pettersen et al., is reference 22.

1. Lorenzen, E., Dodig-Crnkovic, T., Kotliar, I. B., Pin, E., Ceraudo, E., Vaughan, R. D., Uhlen, M., Huber, T., Schwenk, J. M., and Sakmar, T. P. (2019) Multiplexed analysis of the secretin-like GPCR-RAMP interactome. *Sci Adv* **5**, eaaw2778

2. Jumper, J., Evans, R., Pritzel, A., Green, T., Figurnov, M., Ronneberger, O., Tunyasuvunakool, K., Bates, R., Zidek, A., Potapenko, A., Bridgland, A., Meyer, C., Kohl, S. A. A., Ballard, A. J., Cowie, A., Romera-Paredes, B., Nikolov, S., Jain, R., Adler, J., Back, T., Petersen, S., Reiman, D., Clancy, E., Zielinski, M., Steinegger, M., Pacholska, M., Berghammer, T., Bodenstein, S., Silver, D., Vinyals, O., Senior, A. W., Kavukcuoglu, K., Kohli, P., and Hassabis, D. (2021) Highly accurate protein structure prediction with AlphaFold. *Nature* **596**, 583-589

3. Evans, R., O’Neill, M., Pritzel, A., Antropova, N., Senior, A., Green, T., Žídek, A., Bates, R., Blackwell, S., Yim, J., Ronneberger, O., Bodenstein, S., Zielinski, M., Bridgland, A., Potapenko, A., Cowie, A., Tunyasuvunakool, K., Jain, R., Clancy, E., Kohli, P., Jumper, J., and Hassabis, D. (2021) Protein complex prediction with AlphaFold-Multimer. *bioRxiv*, 2021.2010.2004.463034

4. Cao, C., Kang, H. J., Singh, I., Chen, H., Zhang, C., Ye, W., Hayes, B. W., Liu, J., Gumpper, R. H., Bender, B. J., Slocum, S. T., Krumm, B. E., Lansu, K., McCorvy, J. D., Kroeze, W. K., English, J. G., DiBerto, J. F., Olsen, R. H. J., Huang, X. P., Zhang, S., Liu, Y., Kim, K., Karpiak, J., Jan, L. Y., Abraham, S. N., Jin, J., Shoichet, B. K., Fay, J. F., and Roth, B. L. (2021) Structure, function and pharmacology of human itch GPCRs. *Nature* **600**, 170-175

5. Isberg, V., de Graaf, C., Bortolato, A., Cherezov, V., Katritch, V., Marshall, F. H., Mordalski, S., Pin, J. P., Stevens, R. C., Vriend, G., and Gloriam, D. E. (2015) Generic GPCR residue numbers - aligning topology maps while minding the gaps. *Trends Pharmacol Sci* **36**, 22-31

6. Pandy-Szekeres, G., Munk, C., Tsonkov, T. M., Mordalski, S., Harpsoe, K., Hauser, A. S., Bojarski, A. J., and Gloriam, D. E. (2018) GPCRdb in 2018: adding GPCR structure models and ligands. *Nucleic Acids Res* **46**, D440-D446

7. Krissinel, E., and Henrick, K. (2007) Inference of macromolecular assemblies from crystalline state. *J Mol Biol* **372**, 774-797

8. Goddard, T. D., Huang, C. C., Meng, E. C., Pettersen, E. F., Couch, G. S., Morris, J. H., and Ferrin, T. E. (2018) UCSF ChimeraX: Meeting modern challenges in visualization and analysis. *Protein Sci* **27**, 14-25

9. Pettersen, E. F., Goddard, T. D., Huang, C. C., Meng, E. C., Couch, G. S., Croll, T. I., Morris, J. H., and Ferrin, T. E. (2021) UCSF ChimeraX: Structure visualization for researchers, educators, and developers. *Protein Sci* **30**, 70-82
